# Psychometric Validity of the Areas of Work Life Scale (AWS) in Teachers and Healthcare Workers in México

**DOI:** 10.3390/ejihpe13080111

**Published:** 2023-08-15

**Authors:** Arturo Juárez-García, César Merino-Soto, Javier García-Rivas

**Affiliations:** 1 Centro de Investigación Transdisciplinar e Psicología, Universidad Autónoma del Estado de Morelos, Cuernavaca Morelos 62350, Mexico; 2 Instituto de Investigación de Psicología, Universidad de San Martin de Porres, Lima 15048, Peru; sikayax@yahoo.com.ar; 3 Centro Interamericano de Estudios de Seguridad Social (CIESS), Ciudad de Mexico 10200, Mexico; jgarciar@uci.edu

**Keywords:** AWS scale, psychosocial stressors, burnout predictors

## Abstract

The areas of work life scale (AWS) has shown to be a suitable marker of perceived fit between employees’ abilities and the psychosocial demands of the job, but validation studies are practically nonexistent in the Latino population. The purpose of this study was twofold: firstly, to examine the factor structure, reliability, and invariance between sex and occupation of the AWS scale, and secondly, to test the AWS–burnout relationship within the framework of the structural mediational model proposed by Leiter and Maslach (2005). N = 305 health workers and N = 324 teachers from different work settings answered the AWS and MBI-GS scales. In this study, 64.4% of the participants were females (N = 405), and the mean age was 34.7 (sd = 11.7, rank = 56). Robust methods for statistical analyses were used. The results showed that the original version had marginal fit indices due to a method effect (negative phrasing items), and when seven negative items were removed, a final best model was found (CFI = 0.997; RMSEA = 0.060; SRMRu = 0.047). Non-invariance between occupation and sex was found, and the internal consistency was from marginal to satisfactory (ω = 0.658 to 0.840). The mediational structural model tested confirmed the expected associations between AWS and burnout. In conclusion, the Mexican translation of the AWS in its 22-reduced version showed reliability and validity in Mexican work contexts, specifically in healthcare workers and teachers.

## 1. Introduction

According to the World Health Organization (WHO), 15% of working-age adults were estimated to have a mental disorder in 2019, and it is estimated that 12 billion working days are lost every year globally due to depression and anxiety at a cost of USD 1 trillion per year in lost productivity [1]. Before the COVID-19 pandemic, one in every eight people (i.e., 970 million people around the world) were living with a mental health disorder, with anxiety and depressive disorders being the most common [2]. The pandemic has led to a 27.6 increase in cases of major depressive disorder and a 25.6% increase in cases of anxiety disorders worldwide in 2020 (up to 288 and 384 million people, respectively). Together, these ailments have caused an average of about 260 additional disability-adjusted life years (DALYs) per 100,000 population [2].

Specific occupational groups have worked at an increased risk of mental health problems during the COVID-19 pandemic. According to some recent systematic reviews, frontline healthcare workers suffered from several mental health problems, including anxiety, depression, fatigue, stress, sleep disturbances, psychiatric symptoms, suicidal ideation, and particularly, burnout syndrome [3,4,5,6]. To a lesser extent, but as important contributors to society, teachers at different educational levels have also experienced adverse psychological symptomatology (stress, depression, anxiety, and burnout syndrome) during the COVID-19 pandemic according to other studies [7,8].

Although the conception of burnout syndrome is still subject to some debate [9,10] and its definition varies across authors, regions, and contexts, a 35-year review [11] concludes that a three-dimensional definition has achieved almost universal acceptance in research. So, burnout is a specific type of chronic workplace stress-related outcome characterized by feelings of exhaustion, cynicism towards the job, and a sense of ineffectiveness [12]. Lately, burnout has drawn attention for three main reasons: (1) it is considered a major pathogenic mediator between job conditions and several health problems [13], (2) some systematic reviews have shown increasing rates of burnout as an aftermath of the pandemic [2,5], and (3) it is now recognized in the International Classification of Diseases (ICD-11) as a “problem related to employment or unemployment” [14].

Risks of burnout syndrome and mental health in the workplace have been termed as “psychosocial risk factors”, and involve different characteristics of job content, work organization/schedule factors, social relationships at work, and other several specific features of work conditions [15]. Recent findings from ILO reveal that working conditions and employment quality during and in post-pandemic times have been deteriorating in developing countries, particularly in Latin America [16], so research on psychosocial risks and burnout in the region is highly needed.

To better understand how the organizational context affects a worker’s well-being (inherent to the fundamental concept of burnout), we need to recognize the relationship between the individual and the situation. Most psychosocial risk factors and job stress models agree that chronic stress (e.g., burnout) results from an imbalance between demands and the worker’s conditions or job resources to cope with them [17,18,19]. Recent international systematic reviews and metanalyses have shown that the main predictors of occupational burnout are related to job demands (e.g., workload), conflicting relationships at work (e.g., low social support and aggression), lack of job control (or autonomy), low rewards (e.g., sense of being unfairly treated) and bureaucratic limitations, being the most important [20,21,22,23,24,25].

**Figure 1:**
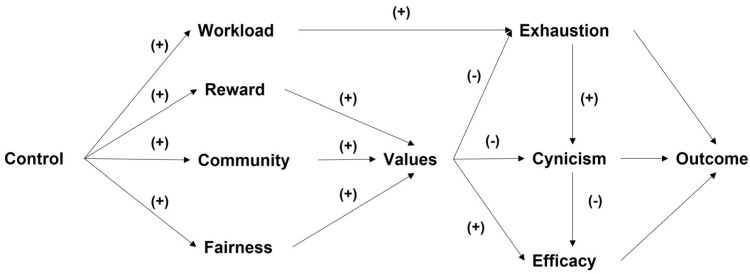
Mediational model of job burnout (Source: Leiter and Maslach, 2004 [30]). Negative or positive expected relationships are marked in the model. Depending on the outcome, directions are expected.

Leiter and Maslach [26] proposed that a gap or mismatch between the person and the job is the main cause of burnout. They argue that such discrepancies arise when there are unresolved organizational problems or job changes that have left out critical psychological needs or capacities of workers, a situation that they find unacceptable. Thus, the authors emphasize that organizational conditions are not the critical issue but it is the extent to which these job demands or work characteristics are consistent with workers’ expectations that is crucial. Maslach [27] defined six areas of work life in which a mismatch between job psychosocial conditions and the worker can occur: workload (when employees must do too much work), control (not being able to make choices in one’s job), rewards (poor salary, benefits, recognition, etc.), community (low connection with others in the workplace), fairness (weak system of justice and unfair processes), and values (discrepancy between personal and organizational or workmate values). These psychosocial stressors coincide with predictors of occupational burnout found in recent systematic reviews mentioned above. It is important to note that the authors consider burnout as a negative outcome of the mismatch in these six areas, and engagement as a positive psychological effect when there is an adequate fit between work and worker. Thus, in this model, burnout is a potential pathogenic mediator between the organizational context and several other negative (or positive) outcomes or changes, which is why the model is called “the mediational model of job burnout” [28] (Figure 1).

The Areas of Work Life Scale (AWS) was created by Leiter and Maslach [29,30] as a 29-item measure to produce distinct scores for each of these six areas: workload (6), control (3), reward (4), community (5), fairness (6) and values (5). The items were worded as statements of perceived congruence or incongruence between oneself and the job. It emerged from qualitative analyses based on written comments provided by hospital workers, and from a series of staff surveys conducted by authors as a means of assessing the constructs underlying the analysis of the six areas of work life [30].

AWS dimensions have demonstrated to be consistent predictors of burnout and important targets for prevention in different regions and sectors in recent studies [31,32,33], so the mediational model of burnout, which deals with a model extended to its predictors from the areas of work life, is a modern and promising line of research that needs to be further explored, especially in the post-pandemic times in different cultures [34,35].

As for the AWS validity and psychometric evidence, a seminal study yielded a theoretically consistent factor structure of six dimensions and consistently showed cross-sectional and longitudinal correlations with burnout scales in the USA, Canada, Finland, and Italian samples [30], which was later replicated in Spain [36], Germany [37], Japan [38], Vietnam [39] and Poland [40]. However, some limitations can be identified in most of the above-mentioned studies: (1) Most psychometric approaches only use Exploratory Factor Analyses (EFA) with the Kaiser rule criterion [41] (principal components method, eigenvalues equal to or greater than 1, and Varimax rotation), which is currently the least recommended method to validate constructs [42,43]. (2) Confirmatory Factor Analyses (CFA), a more robust technique, is used in some of these studies; however, they were used with the maximum likelihood (ML) function, which is very sensitive to the breach of the multivariate normality assumption, and in this case, it miscalculates the estimated parameters of the items and latent covariance [44]. (3) Invariance of AWS across groups is assumed when direct comparisons are carried out (e.g., sex, age, occupation, etc.); however, without testing if such variance may be relevant to the latent variable assessed, a bias could occur, invalidating any differences originally found between groups. Measurement invariance estimation ensures that the meaning and levels that outline the construct are equivalent across groups and can be tested through multigroup confirmatory factor analysis (MGCFA) [45].

It is important to note that the previous validation studies of AWS consistently report low factor loads in some items (λ < 0.40), correlations between some residual’s errors, and marginal reliability coefficients (<=0.70). Given these results, some studies with Spanish translations have suggested reduced versions of the AWS, cutting even more than 60% of the original items of the scale, which we consider calls into question the original content validity of the scale [46]. Hence, psychometric research on AWS still a fertile field.

The study by Dominguez and colleagues in Peruvian teachers [47] is the only study found in Latin America, and it uses the exploratory structural equation modeling (ESEM) technique, a more flexible and comprehensive option that incorporates the advantages of both the EFA and CFA techniques. This study could not confirm the original and expected item and dimension structures of the AWS and showed the presence of a method factor associated with reverse-coded items. Like some other previous findings, it observed low factor loadings and low-reliability coefficients in some dimensions. Furthermore, measurement invariance was not tested.

Given the above, is clear that the psychometric evidence of AWS still scarce worldwide and practically non-existent in Latin America. More research on the psychometric properties of AWS in different occupations in Latin America is highly needed. Thus, the purpose of this study is to examine the psychometric properties of the AWS scale using a CFA approach (factor structure and reliability), and the multigroup invariance (MGCFA) between sex and occupations in a Mexican sample of health workers and teachers. Additionally, as evidence of validity with external variables, we tested the structural relationships between AWS and burnout (MBI) within the framework of a partial mediational model proposed by Maslach.

## 2. Materials and Methods

### 2.1. Participants

A total of N = 629 employees (N = 324 teachers at different levels of teaching and N = 305 health workers, mainly doctors and nurses) from the public sector of different organizations in Mexico participated voluntarily (non-random participant-driven sampling). In this study, 64.4% of the participants were females (N = 405), 48.8% were married or lived with a partner (N = 307), and the rest were single or divorced. The mean age was 34.7 years old, (sd = 11.7, rank = 56) and no significant differences were found between males’ and females’ ages (*p* = 0.32).

An occupational sample of health workers and teachers was chosen because of their vulnerability to burnout and psychosocial demands at work and, both are in human service organizations, so they are well suited to analyze the differential functioning of the AWS scale between the two occupations. The response rate was 80% for both groups. Efforts were made to respect at least a 5:1 item/participant ratio as suggested by some authors [48]. The socio-demographic profile was moderately similar between teachers and health workers for sex (χ2 = 23.16, gl = 1, *p* < 0.01, VCramer = 0.19), age (Mann–Whitney W = 71,072, *p* < 0.01, r = 0.43), and marital status (χ2 = 89.66, gl = 4, *p* < 0.01, VCramer = 0.37).

### 2.2. Measures

#### 2.2.1. Six Areas of Work Life Scale (AWS)

The original English version of the Areas of Work Scale© was adapted and translated into Spanish with the authorization of its authors and Mind Garden publisher [29,49]. This version consists of 29 items organized into five dimensions as follows: workload (6 items), control (3 items), reward (3 items), community (5 items), justice (6 items), and values (5 items). These are rated on a Likert-type scale that reflects the intensity of agreement with the situation described in the item and has five levels ranging from 1 (strongly disagree), through 3 (difficult to decide), to 5 (strongly agree). The score for some items was reversed (items 1, 2, 3, 4, 12, 13, 18, 23, 24 and 29) according to the manual.

The translation process was carried out according to international recommendations for the cross-cultural adaptation of self-reports [50,51]. Firstly, according to the back-translation procedure, a translation from English to Spanish was carried out and then the re-translation from Spanish to English was carried out separately by two specialists (with the mother tongue according to the translation stage); discrepancies were reviewed with the help of a third specialist until it was adjusted to a single final Spanish version. In the next step, this version was sent to a review committee of 5 Latin-American experts for its final review and adjustment, who were chosen mainly for their experience in the assessment of burnout and psychosocial factors. The committee analyzed the semantic understanding of each item until a version was agreed upon.

#### 2.2.2. Maslach Burnout Inventory General Survey (MBI-GS)

It was developed in 1996 by Schaufeli, Leiter, Maslach, and Jackson [52] from the original version of the MBI Human Services Survey (MBI-HSS). It was designed to evaluate burnout in any occupation and according to the same authors, it has greater theoretical soundness and better psychometric indicators than the original scale (MBI-HSS). Originally, it consists of 16 items organized in three dimensions as follows: emotional exhaustion (AE), 5 items; cynicism (CY), 5 items; and professional efficacy (PE), 6 items. These are assessed on a Likert-type scale that reflects the frequency with which workers experience the situation described in the item and has seven degrees ranging from 0 (never) to 6 (every day). The validated Latin-American version of 15 items was used (without item 13 due to problematic psychometric performance) [53]. As for the structural validity in this study, the fit indices were satisfactory: WLSMV-X2 = 402.606 (*p* < 0.01, df = 87), CFI = 0.991, RMSEA = 0.079 (90% CI = 0.071, 0.087), SRMRu = 0.041 (se = 0.001), and close fit Z-SRMRu = −10.02 (*p* = 1.0). The parameters at the item level and inter-factorial correlations are reported in Appendix A.

### 2.3. Procedures

#### 2.3.1. Ethical Considerations

All subjects provided their informed consent for inclusion before they participated in this study. This study was conducted following the Declaration of Helsinki [54], and the research protocol was approved by the Ethics Committee of the Center for Transdisciplinary Research in Psychology of the Autonomous University of the State of Morelos (Universidad Autónoma del Estado de Morelos, UAEM) under code 161220-50.

#### 2.3.2. Analysis and Treatment of Potentially Biased Responses and Missing Values

Biased responses were analyzed because of the probable missing/careless responses and considering the variability of the response pattern associated with the content of each dimension of the AWS. The long string index (LS) was implemented [55]. LS identifies the longest sequence of identical responses within the analyzed set of items. The cutoff point was set at the median plus the third quartile, which was verified by observing the frequency distribution of the repeated patterns.

Missing values were evaluated to detect whether they were missing completely at random (MCAR) or not [56]. Data with an MCAR pattern are considered before applying simple or modeling imputation strategies. The X^2^ MCAR test was used, in which a statistically significant result (*p* < 0.01) fails to reject the null hypothesis of MCAR in the data. Due to the sensitivity of the normality assumption linked to MCAR-X2 [56], this global test was supplemented at the item level with a regression-based approach [57]. The R programs naniar [58] and RBtest [57] were implemented. Finally, the imputation of the missing data was carried out using an ordinal logistic regression approach [59]. The R program TesDataImputation was used [60].

#### 2.3.3. Item Analysis

As a contribution to the content validity of the items [61], descriptive statistics were reported; among them were the normality test with the Anderson–Darling test [62], and association coefficients with demographic variables sex, age, and type of profession (Glass rank biserial, Spearman, and Intraclass Correlation Coefficient 1 (ICC1), respectively).

#### 2.3.4. Internal Structure

##### Measurement Models

First, the item–dimension relationship was examined using confirmatory factor analysis. Due to a stronger rationale for testing a multidimensional model with five related dimensions, the modeling started with this assumption in contrast with the evaluation of the one-dimensionality and orthogonality of the dimensions performed in previous studies [26,47]. Three main models were tested. The first one was with the items distributed in their expected theoretical dimensions, and with the recoded items (1, 2, 3, 4, 12, 13, 18, 23, 24 and 29). This model represents the original one proposed by Leiter and Maslach [26], in which the dimensions are correlated (CFA-full). In the second model, along with the six dimensions, a method factor was added to account for the possible variance coming from the phrasing of the recoded items. In this model, workload items 5 and 6 were defined as contributors to method variance by negative phrasing, because these items were phrased in the same orientation as items 12, 13, 18, 23, 24, and 29., i.e., these items were oriented toward organizational misfit. This model reevaluated the CFA findings of Domínguez et al. [47], who implemented this same model (CFA-met). To advance this study, a final third model was developed, without the recoded items. This was a shorter measure with items phrased in only one direction, and the absence of method effects associated with the negative phrasing of the items (CFA-nneg). This third model represented an abbreviated version based on the quality of the parameters obtained and partially represented the model of Masluk et al. [46], who developed an abbreviated version, but with some different items.

The modeling used the weighted least squares with means and variances adjusted estimator (WLSMV), which is recommended for modeling categorical variables and obtaining more accurate factor loadings and inter-factorial correlations [63] The fit of each model was evaluated with two approaches: (a) globally, with tests of exact fit and approximate fit, and (b) locally, observing the magnitude of the factor loadings and residuals. In the global fit, the exact fit tests were χ^2^ goodness-of-fit (WLSMV-χ^2^) and SRMR-unbiased (SRMRu), used as hypothesis testing with confidence intervals [64]. SRMRu for these decisions has shown to have lower Type I error and has proven to be more robust than different estimation methods [64,65,66] on non-normal data, especially with estimators such as WLSMV, and with sensitivity to misspecifications due to omitted cross-loadings [67]. With SRMRu, the fit was evaluated with an adjusted cutoff point based on the ratio SRMRu/${\bar{R}}^{2}$: ${\bar{R}}^{2}$ × 0.05 (${\bar{R}}^{2}$ × 0.05) (=average squared factor loadings, or communality) [68]. In the local setting, the magnitude of the reported loadings was observed at three levels: low, medium, and high (0.40, 0.60 and 0.80, respectively) [65]; the model specification errors (residual correlations) [64] were observed at three severity levels: |0.10|, |0.20| and |0.30| [68].

##### Measurement Equivalence

The sex and occupation groups were chosen to study the equivalence of the measure because they can both be associated with differences in the intensity or structure of the work factors. A strategy for assessing the degree of non-equivalence was used, using two effect size indices. The first is used in the SEM framework, *dMACS* [69], to estimate the degree of bias due to non-invariance. This is expressed as the standardized mean difference for each item between the compared groups produced by differences in factor loadings and intercepts, and is interpretable as Cohen’s *d* coefficient. In agreement with Nye et al. [69], the cutoff points for interpreting *dMACS* were 0.40, 0.60 and 0.80 (small, medium, and large, respectively), concerning their effect on the measures. The second strategy that emphasized the effect size of possible non-invariance was ordinal logistic regression [70]. This procedure estimates three hierarchical models of the predictors of effect on item responses: effect from the attribute, from the group, and the interaction between the attribute and the group (DIF1, DIF2, and DIF3, respectively). The DIF tests (Δχ^2^) first assessed the presence of some type of DIF (Δχ^2^ DIF3-DIF1); if this result reached statistical and practical significance, the specific type of DIF was assessed: non-uniform (Δχ^2^ DIF3-DIF2) and uniform (Δχ^2^ DIF2-DIF1). The criterion for statistical significance was *p* < 0.01; the criterion for practical significance was the Nagelkerke-R^2^ difference of each model compared (ΔR2) at three levels [71]: trivial (<0.035), moderate (≥0.035), and large (≥0.070).

##### Reliability

Modeling-based reliability estimates were obtained through the omega coefficient. The omega coefficient (ω) is a more appropriate indicator than the traditional alpha Cronbach (α); it can better represent the reliable variance of the construct and has an advantage in that problems associated with inflation and attenuation of internal consistency estimation are far less likely to occur. Acceptable levels of omega must be above 0.70 and some studies have pointed out it as the most promising alternative to measure the reliability of a test [72].

#### 2.3.5. Association with Variables and Partial Mediational Model

First, the association and linear regression of the AWS with the constructs measured using MBI-GS (i.e., emotional exhaustion, professional efficacy, and cynicism) was explored. Second, based on the replicability of the multi-variate structural relationships between the variables of the AWS and MBI-GS as proposed in the mediational model by Leiter and Maslach [28] (Figure 1), although the estimated mediational model was partial, there was no outcome variable in the model for this study.

After establishing the fit of this structural model, its invariance between sex and occupation groups was also estimated. First, a global evaluation was used, comparing the fit between the model with and without restrictions of equality of path coefficients between groups. If the overall test indicated not to accept the null hypothesis of equality, the second step was to examine the source of the differences. Each difference was examined with the Wald z test, at the *p* < 0.003 level (0.05 with Bonferroni adjustment for the 14 regression coefficients compared: 0.05/14).

## 3. Results

### 3.1. Preliminary Analysis

#### 3.1.1. IE/C Responses

Median = 5 and Q3 = 6; cutoff point: 10 consecutive equal response patterns. Thirty-eight cases were detected (6.04%). These cases were removed, with 591 cases remaining for analysis.

#### 3.1.2. Missing Values

The MCAR test resulted in X^2^ = 1259 (*p* = 0.00001), with 42 distinct patterns of missing responses. The number of missing values on the items ranged from 1 (0.16%, item) to 10 (1.6%, item), with M = 4.8 (0.8%; Md = 5), and SD = 1.9 (0.3%). However, at the item level, each item was identified as MCAR data. Therefore, we proceeded with imputation, and the complete data were analyzed in the next phase.

### 3.2. Item Analysis

The items did not maintain multivariate normality (Henze–Zirkler test = 1.40, *p* < 0.01), nor univariate normality (Median = 29.15, Min = 19.38, Max = 43.49). Table 1 shows the results, in which the mean response of the positive and negative items seems to be indistinguishable. Skewness (<|1.0|) and kurtosis (<|1.3|) did not exceed values beyond |1.5|. As for the association with other variables, low associations were observed with age (Min = 0.025, Max = 0.011) and occupation (health personnel vs. teachers: Min = 0.0 , Max = 0.08), but higher associations with sex (Min = 0.22, Max = 0.27). Predominantly, these associations were not statistically significant (Table 1).

### 3.3. Internal Structure

#### 3.3.1. Dimensionality

Table 2 shows the fit results and item parameters of all the models tested. The first three models, corresponding to the full model (CFA-full), model with method factor (CFAmet), and short version model without recoded items (CFA-nneg), yielded different conclusions. The CFA-full showed a fit that was not acceptable and above the fit criteria (SRMRu > 0.040); the model with method factor (CFA-met) provided a superior fit compared with CFA-full, and was not statistically significant, but was not below the fit criteria (SRMRu > 0.043). The CFA-nneg model showed better coefficients (CFI < 0.998, RMSEA < 0.066, SRMRu < 0.048), with a non-statistically significant fit. In this model, negative items 12 and 13 were not removed in order to avoid an extreme reduction in the content and extent of the factor; instead, it was decided to remove only item 13. We chose this item to reduce the possible effect of the presentation of consecutive items in the AWS.

In the observation of the tested models, item 20 (from FAIR dimension) was of consistently low factor loading (<0.40). Therefore, we tested a new fourth model without this item to see the possible improvement in the fit. However, the improvement was not substantial, and the item was not removed. In conclusion, the model where most of the negative items (CFA-nneg-) were removed was the accepted model (AWS 22 items-reduced version).

#### 3.3.2. Inter-factorial Correlation

As can be seen in Table 2, all latent correlations were statistically significant.

#### 3.3.3. Reliability

In the modeling, the omegas coefficients were generally above ω = 0.60 (Table 3). In the final model (AWS without negative items), the trend was to obtain reliabilities around or greater than 0.70, except for the control (CL) score. There was also an apparent effect of the number of items on the magnitude of the reliability because in the final model: the dimensions with 4 items tended to show higher reliabilities than the dimensions with 3 items. The omega reliability of FAIR decreased substantially after removing item 20. This effect reinforced the decision to retain it even though its factor loadings were low.

#### 3.3.4. Measurement Equivalence

In the sex group (Table 4), the magnitude of invariance in *dmacs* (Md = 0.19, Min = 0.02, Max = 0.32) and logistic regression (DIF3-DIF1: R2 Md = 0.001, Min = 0.00, Max = 0.006) remained at a trivial level of impact on each item, and none was statistically significant at the *p* > 0.01. Similarly, this also occurred in the occupation groups for the *dmacs* (Md = 0.14, Min = 0.01, Max = 0.35) and ordinal logistic regression (DIF3-DIF1: R2 Md = 0.004, Min = 0.00, Max = 0.024) methods, in which a consistently trivial level of impact was observed for all items. Although some statistical differences were observed in this group in the DIF3-DIF1 comparison, the size of these differences was trivial. In conclusion, the AWS metrics can be considered acceptably invariant in the groups based on the sex and occupation of the participants. The complete results are shown in Table 4.

### 3.4. Association with Variables and Partial Mediation Model Testing

Table 5 shows the dimensions of the AWS and their correlations with burnout indicators. All show theoretical consistency, and they have between moderate to high coefficient sizes. The control (CL) dimension had the highest covariance with the rest of the dimensions. On the other hand, the latent inter-factor correlations between the AWS and MBI-GS dimensions observed a pattern of consistently theoretical directions. The magnitude of the correlation between exhaustion and WL was the highest (r = 0.680) among the correlations outcomes (MBI-GS).

**Figure 2:**
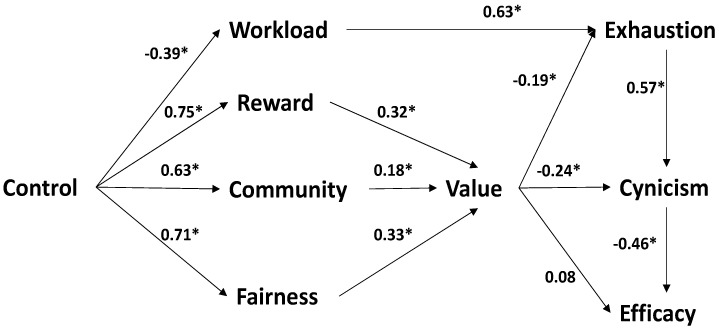
Structural modeling results of the partial mediational model of burnout (AWS and burnout relationships). * *p* < 0.05.

In Figure 2, the structural model obtained an acceptable initial fit (MLR X2 = 1392.932, df = 616, *p* < 0.01; SRMR z = 0.988, *p* = 0.162), due to the discrepancy between the CFI = 0.888 and the rest of the fit indicators: RMSEA = 0.047, 90% CI = 0.044, 0.050; SRMR = 0.068; SRMRu = 0.054, 90% CI = 0.047, 0.061). The positive or negative direction of the beta coefficients between the constructs exactly reproduced the expected direction (see Figure 1). All coefficients were statistically significant except the values → efficacy association. The model was recalculated but with the values → efficacy path at a value of 0, obtaining a non-significant difference between the two models: X2 = 1.52 and *p* = 21. Therefore, the accepted structural model included the values → efficacy path, for further evaluation of its invariance.

#### Invariance of the Partial Mediation Model

With the model identified, its structural invariance was evaluated, and as in the measurement invariance, the groups compared were sex and occupation. In the sex group, the comparison between the model with free estimation (MLR-X2 = 2177.18, df = 1232, *p* < 0.01) and with restriction of the regression coefficients (MLR-X2 = 2022.36, df = 1245, *p* < 0.01) was statistically significant (X2 = 34.41, df = 13, *p* = 0.001). Analyzing the source of this discrepancy (Table 6, headed sex group), the largest contrast occurred in CYN → EFF, WL → EFF, and WAL → EFF associations. However, these were not statistically significant (*p* > 0.003 [Wald z test correction]).

In the occupation group (health workers and teachers), the comparison between the model with path coefficient-free estimation (MLR-X2 = 2530.81 (df = 1232), *p* < 0.01), versus its equality-constrained estimation (MLR-X2 = 2567.405, df = 1245, *p* < 0.01) yielded a statistically significant difference (X2 = 34.41, df = 13, *p* = 0.001). Analyzing the source of this discrepancy (Table 6, heading occupation group), the largest difference (Δ_z_) was in REW → VAL association and COM → VAL association, but these were not statistically significant (*p* > 0.003 [Wald z test correction]); similarly, none of the other contrasts were detected as statistically significant.

## 4. Discussion

The purpose of this study was twofold: firstly, to examine the factor structure, reliability, and sex and occupation invariance of the AWS scale, and secondly, to test the AWS–burnout relationship within the framework of the structural mediational model proposed by Leiter and Maslach, testing in turn its invariance.

As for the general psychometric properties of the AWS scale, CFA modeling was performed, in agreement with previous studies [36,37,46,73]; however, this study used more robust methods, such as WLSMV, unbiased local estimators (e.g., SRMRu), omega coefficient (ω) for reliability, and effect size coefficients of invariance (e.g., dMACS), among others. Likewise, the scope of this study was larger than previous studies, incorporating evidence of the invariance of AWS dimensions and its structural relationship with burnout syndrome.

The translated original full version of the AWS in a sample of two occupations of Mexican workers showed poor to marginal fit indices (CFI = 0.944; RMSEA = 0.077; SRMRu = 0.058). Although a “method factor” was found (because of reverse coding in negative items), the superior fit was in the short version when most of the negative phrasing items were removed (7 items) (CFI = 0.997; RMSEA = 0.060; SRMRu = 0.047), so we considered this one as the accepted final model (22 items). The decision to remove the negative items had direct support from previous studies with the AWS, in which a short version was developed excluding these items [46] and the method effect produced by them [47]. Indirectly, it was also supported by the consistent methodological literature which has shown the deterioration effects of a measurement model when items in opposite directions are used (reversed or negatively worded items relative to the construct) [61,74,75].

As in the seminal validity study of AWS and other studies [30,39,47], item 20 of the *fairness* dimension (“*Opportunities are decided solely on merit*”) had the lowest factor loading; however, it did not produce substantial changes in the global fit. In order to not reduce the content of this dimension, the item was not removed from the final model. This decision balanced the content of the instrument and the consequences on the AWS modeling.

Reliability coefficients among the different tested models were very similar. In the final AWS model (AWS-no neg), the coefficients were from marginal to satisfactory (ω = 0.658 to 0.840; Table 3). Workload (WL) and fairness (FAIR) dimensions had the lowest coefficients. However, rating the reliability of the scores of some instruments may depend less on a generic benchmark than on other attributes such as the intended context of use of the instrument, the conceptual breadth assessed, the number of items, the purpose of use, etc. Because the AWS measures variables of the work context, and its use is predominantly intended to describe groups of workers exposed to these variables as a screening tool, the level of reliability of the AWS obtained may be sufficient. In this sense, the results have an important implication: in the psychometric evaluation of the AWS, the removal of items will have effects on reliability, and the researcher must weigh this effect (i.e., decrease or increase) against the item’s content validity.

An important finding was that AWS metrics can be considered acceptably invariant in the groups based on the sex and occupation of the participants. Although some items have tendencies to non-equivalence, the effect sizes were trivial. Regarding the investigation of the structural model between areas of work life and burnout (AWS → MBI-GS), predictive weights and directions of the relationships were consistent with the theory and with the findings of Leiter and Maslach [26,28], because the only statistically non-significant association was VAL → EFF. When examining the invariance of this parameter, this relationship was slightly discrepant between sexes and occupations, but within sampling error. Therefore, this lack of association does not detract from the overall findings of the scale. The invariance of this model was examined and, although the overall test of difference yielded statistical significance, the specific source was not statistically detected. Application of the Bonferroni adjustment reduced this occurrence of false positives.

The results of the analysis, although not established as a primary study objective, suggest that the detection and treatment of responses with possible IE/C should be implemented a priori. The detected prevalence of IC/C can be considered low and is within the prevalence found in the previous literature [76], but slightly higher than the infrequent literature addressing this issue in the study of psychosocial factors [61].

One limitation is that the method of identifying IE/C and its cutoff point is conditional on the subjective decision of the researcher, so a variation in both may indicate different amounts of detected responses. In contrast, the method chosen is more appropriate and simpler to implement when a multidimensional measure, and was considered sufficient for this study.

Another limitation was that the modeling method (CFA) omitted the estimation of cross-loadings. In this regard, another type of modeling, such as ESEM, is more effective in detecting them without leaving the SEM framework. As stated above, the magnitude and number of cross-loads in the present study, as well as their effective impact on the modeling performed, are unknown. Even with this omission of cross-loadings, the overall fit coefficients of the AWS measurement models were not substantially low, and only the introduction of the second model (method factor, CFA-met) was sufficient for the fit to be satisfactory (i.e., statistically non-significant using SRMRu as a criterion). Overall, we can assume an innocuous effect of the potential cross-loadings on the AWS, and that they were not detected with the adjustment indices due to their small magnitude [67]. On the other hand, the constraints of self-reporting measures and the use of a non-representative sample must also be recognized as limitations, but we believe that the robust estimations used in this study attenuated these limitations to some extent.

### Future Research Directions

This study contributed to the evidence of the psychometric properties of AWS in a cultural context different from where it was developed, and it analyzed its viability to be used reliably as a tool for the evaluation of psychosocial factors in the organizational context of Mexican workplaces. However, more studies are needed to explore the psychometric properties of the AWS in informal workers, white-collar and blue-collar workers, and other occupations. Also, it is important to continue analyzing its invariant properties across different cultures, or simply across different timepoints. This last point is particularly relevant as more longitudinal studies are also needed to confirm the convergent validity relationships of the AWS dimensions with burnout and other health outcomes.

Although the present study focused specifically on the psychometric properties of the AWS and its dimensions, research is still needed to address other dimensions specific to different contexts and cultures that cause burnout syndrome due to work/person mismatch. This would expand or enrich the AWS scale and make it more universal. For instance, job crafting attitudes, specific demands, and specific resources at work within occupations and sectors from a flexible and heuristic view [77,78]. Likewise, beyond three burnout dimensions, new latent profiles of burnout have emerged conceptually (overextended, ineffective, disengaged, etc.), so it seems that the mediational model of burnout also needs to expand and consider these new multidimensional characteristics of the phenomena [79,80].

Finally, one of the most important aspects of the findings in the present study is related to the reversed or negatively worded items in the original AWS and the possible method effect that it entails. The consistent body of literature on the topic and the clear findings of the present study suggest that AWS (and perhaps any other psychosocial measures) should reevaluate introducing negative items in the measurement of a construct. Unless these negative items are used as measures of bias, these items will tend to generate invalid responses. Although this practice of incorporating opposite items of the construct to avoid acquiescence bias is relevant, it seems that the psychometric cost is higher than the earnings, and the validity of the measurement is compromised. Further studies are needed to explore this method’s effect on the AWS in other samples.

## 5. Conclusions

Given the above, we can conclude that the scores of the AWS in its use for the evaluation of psychosocial factors in Mexican work contexts, specifically in healthcare workers and teachers, seem to be valid and acceptably reliable in a smaller version (20–22 items), eliminating items with negative phrasing that confirm a method effect. Also, for the first time and according to the estimates carried out in a Latin-American sample, AWS scores showed to be invariant by occupation and sex; thus, direct comparisons of psychosocial dimensions of AWS between teachers and health workers are feasible.

## Figures and Tables

**Table 1:** Descriptive statistics of AWS items and their relationship with sex, age, and occupation.

	M	DE	Skew	Ku	AD	Sex	Age	Occ.
Workload (WL)								
AWS1	3.67	1.16	−0.57	−0.75	31.76	0.198	−0.076 *	0.006
AWS2	2.87	1.26	0.14	−1.17	25.31	0.246	−0.096 *	0.018
AWS3	3.04	1.29	−0.11	−1.24	26.28	0.068	−0.052	0.015
AWS4	3.28	1.30	−0.37	−1.07	28.18	0.201	−0.042	0.039
AWS5r	3.45	1.17	−0.56	−0.65	31.22	0.154	−0.003	0.001
AWS6r	3.18	1.30	−0.22	−1.15	23.43	0.275	−0.036	0.028
Control (CL)								
AWS7	3.92	0.96	−0.89	0.50	37.09	0.229	0.0651	0.00
AWS8	3.00	1.28	−0.28	−1.05	25.54	−0.144	−0.025	0.01
AWS9	3.61	1.11	−0.74	−0.23	34.67	0.0255	−0.010	0.00
Rewards (REW)								
AWS10	3.35	1.20	−0.51	−0.74	29.15	−0.059	0.045	0.00
AWS11	3.63	1.13	−0.83	−0.00	35.07	−0.134	0.030	0.00
AWS12r	3.49	1.15	−0.42	−0.76	24.63	0.0948	0.025	0.00
AWS13r	3.40	1.14	−0.32	−0.84	24.26	0.0095	0.013	0.00
Community (COM)								
AWS14	3.39	1.07	−0.63	−0.25	32.32	−0.014	0.025	0.003
AWS15	3.73	1.05	−0.88	0.32	35.38	−0.087	0.101 *	0.029
AWS16	3.69	1.03	−0.81	0.14	37.29	−0.046	0.0706	0.00
AWS17	3.57	1.11	−0.75	−0.15	35.37	−0.218	0.117 *	0.00
AWS18r	3.53	1.24	−0.46	−0.90	26.48	0.0771	0.0468	0.004
Fairness (FAIR)								
AWS19	3.25	1.19	−0.36	−0.80	22.64	0.199	0.0432	0.026
AWS20	3.03	1.15	−0.15	−0.80	19.77	−0.015	0.0171	0.009
AWS21	2.99	0.99	−0.27	−0.33	27.04	−0.218	0.0946 *	0.015
AWS22	3.17	1.14	−0.33	−0.76	24.00	−0.034	0.00533	0.012
AWS23r	3.25	1.18	−0.18	−0.88	19.38	−0.001	0.0113	0.081
AWS24r	3.39	1.25	−0.32	−0.97	21.77	−0.126	−0.0115	0.051
Values (VAL)								
AWS25	3.49	1.03	−0.64	−0.14	32.99	−0.202	0.0764	0.001
AWS26	3.68	1.00	−0.76	0.03	39.82	−0.001	0.0573	0.012
AWS27	3.69	0.99	−0.84	0.33	40.11	−0.108	0.0373 *	0.01
AWS28	3.84	1.08	−0.90	0.26	32.97	0.201	0.0593 *	0.004
AWS29r	3.94	1.20	−0.96	−0.11	43.49	−0.015	0.0791	0.056

Note. Skew, Ku: skewness and kurtosis coefficients. AD: Anderson–Darling normality test. * *p* < 0.05.

**Table 2:** CFA parameters of the tested models and fit indicators.

	CFA-Full	CFA-Met		CFA-Nneg	CFA No Items Neg, No 20
	F	F	Met		
AWS1	0.66	0.653	-	0.68	0.68
AWS2	0.41	0.413	-	0.49	0.49
AWS3	0.70	0.705	-	0.77	0.77
AWS4	0.76	0.763	-	0.81	0.81
AWS5r	0.56	0.564	0.008	-	-
AWS6r	0.46	0.457	−0.022	-	-
AWS7	0.70	0.701	-	0.69	0.69
AWS8	0.60	0.601	-	0.61	0.61
AWS9	0.71	0.709	-	0.72	0.72
AWS10	0.79	0.806	-	0.81	0.81
AWS11	0.78	0.798	-	0.80	0.80
AWS12r	0.61	0.449	0.644	0.45	0.45
AWS13r	0.58	0.408	0.649	-	-
AWS14	0.56	0.562	-	0.57	0.57
AWS15	0.83	0.829	-	0.82	0.82
AWS16	0.90	0.903	-	0.91	0.91
AWS17	0.79	0.795	-	0.80	0.80
AWS18r	0.36	0.331	0.255	-	-
AWS19	0.61	0.618	-	0.62	0.62
AWS20	0.19	0.196	-	0.23	-
AWS21	0.46	0.475	-	0.50	0.50
AWS22	0.76	0.770	-	0.76	0.76
AWS23r	0.53	0.478	0.359	-	-
AWS24r	0.50	0.439	0.335	-	-
AWS25	0.68	0.684	-	0.69	0.69
AWS26	0.55	0.556	-	0.57	0.57
AWS27	0.72	0.724	-	0.73	0.73
AWS28	0.82	0.823	-	0.81	0.81
AWS29r	0.26	0.239	0.142	-	-
WMSLV χ^2^	1617.560 **	1229.30 **		601.89 **	566.021 **
(df)	(362)	(354)		(194)	(174)
CFI	0.944	0.961		0.997	0.978
RMSEA	0.077	0.065		0.060	0.062
(90% CI)	(0.073, 0.080)	(0.061, 0.069)		(0.54, 0.65)	(0.056, 0.068)
Close fit *p*	<0.01	<0.01		<0.01	<0.01
uSRMR	0.056	0.052		0.047	0.048
(se)	(0.001)	(0.001)		(0.001)	(0.001)
(90% CI)	(0.054, 0.059)	(0.050, 0.054)		(0.045, 0.049)	(0.046, 0.050)
Criteria for uSRMR fit					
Close	0.020	0.021		0.023	0.024
Acceptable	0.040	0.043		0.047	0.049
Close fit Z	5.855 **	1.48		−2.01	−1.38

Note. CFA-full: CFA model, all items and related factor. CFA-met: CFA model with method factor (items with negative wording). CFA-nneg: CFA model with no negative items. CFA-nneg no 20: CFA model with no negative items, and no item 20. SRMRu: squared root mean residual unbiased. Close fit Z: z test. Criteria fit: used for SRMRu. ** *p* < 0.01.

**Table 3:** Omega reliability scores of the AWS models.

	WL	CL	REW	COM	FAIR	VAL
Full AWS	0.775	0.656	0.792	0.810	0.666	0.722
AWS + MF	0.775	0.656	0.629	0.797	0.633	0.716
AWS-no neg	0.764	0.658	0.699	0.840	0.759	0.766
AWS-neg-i20	0.764	0.658	0.700	0.839	0.624	0.766

Note. WL: workload: CL: control. REW: rewards. COM: community. FAIR: fairness. VAL: values. Full AWS: AWS with all items. AWS + MF: AWS with method factor. AWS-no neg: AWS without negative items. AWS-neg-i20: AWS without negative items and items 20.

**Table 4:** Measurement equivalence-based size effects indexes.

	Sex (Male, Female)						Occupation (Teachers, Health Workers)					
	Standardized Difference		Ordinal Logistic Regression				Standardized Difference		Ordinal Logistic Regression			
	d_MACS_	Nivel	Δχ^2^ DIF3-DIF1	*p*	ΔR2	Nivel	d_MACS_	Nivel	Δχ^2^ DIF3-DIF1	*p*	ΔR2	Nivel
AWS1	0.19	Trivial	1.568	0.45	0.002	Trivial	0.11	Trivial	25.36	0.00	0.024	Trivial
AWS2	0.20	Trivial	6.592	0.03	0.006	Trivial	0.21	Trivial	15.48	0.00	0.014	Trivial
AWS3	0.27	Trivial	0.156	0.92	0.000	Trivial	0.30	Trivial	15.64	0.00	0.011	Trivial
AWS4	0.32	Trivial	0.987	0.61	0.001	Trivial	0.35	Trivial	12.17	0.00	0.008	Trivial
AWS7	0.11	Trivial	1.624	0.44	0.001	Trivial	0.01	Trivial	0.17	0.91	0.000	Trivial
AWS8	0.16	Trivial	2.491	0.28	0.002	Trivial	0.16	Trivial	5.31	0.07	0.004	Trivial
AWS9	0.02	Trivial	3.440	0.17	0.003	Trivial	0.20	Trivial	6.62	0.03	0.005	Trivial
AWS10	0.31	Trivial	3.164	0.20	0.002	Trivial	0.02	Trivial	2.07	0.35	0.001	Trivial
AWS11	0.17	Trivial	1.611	0.44	0.001	Trivial	0.10	Trivial	0.29	0.86	0.000	Trivial
AWS12r	0.19	Trivial	0.423	0.80	0.000	Trivial	0.06	Trivial	3.98	0.13	0.004	Trivial
AWS14	0.10	Trivial	4.080	0.13	0.004	Trivial	0.11	Trivial	16.66	0.00	0.015	Trivial
AWS15	0.29	Trivial	0.445	0.80	0.000	Trivial	0.16	Trivial	8.10	0.01	0.005	Trivial
AWS16	0.32	Trivial	0.401	0.81	0.000	Trivial	0.15	Trivial	0.51	0.77	0.000	Trivial
AWS17	0.29	Trivial	0.610	0.73	0.000	Trivial	0.11	Trivial	5.84	0.05	0.004	Trivial
AWS19	0.15	Trivial	2.537	0.28	0.002	Trivial	0.19	Trivial	0.39	0.82	0.000	Trivial
AWS20	0.15	Trivial	0.411	0.81	0.001	Trivial	0.14	Trivial	0.43	0.80	0.001	Trivial
AWS21	0.15	Trivial	1.012	0.60	0.001	Trivial	0.17	Trivial	1.83	0.40	0.002	Trivial
AWS22	0.29	Trivial	3.882	0.14	0.004	Trivial	0.31	Trivial	9.63	0.00	0.009	Trivial
AWS25	0.13	Trivial	6.907	0.032	0.006	Trivial	0.08	Trivial	1.76	0.41	0.001	Trivial
AWS26	0.15	Trivial	0.837	0.658	0.001	Trivial	0.23	Trivial	17.47	0.00	0.014	Trivial
AWS27	0.27	Trivial	2.622	0.270	0.002	Trivial	0.14	Trivial	5.57	0.06	0.004	Trivial
AWS28	0.30	Trivial	2.086	0.352	0.002	Trivial	0.11	Trivial	1.56	0.45	0.001	Trivial

**Table 5:** Correlations between AWS and MBI-GS dimensions.

	WL (4 Items)	CON (3 Items)	REW (3 Items)	COMM (4 Items)	FAIR (4 Items)	VAL (4 Items)
AWS						
WL	1.0					
CON	−0.397	1.000				
REW	−0.300	0.756	1.000			
COMM	−0.252	0.634	0.479	1.000		
FAIR	−0.282	0.710	0.536	0.450	1.0	
VAL	−0.230	0.580	0.568	0.477	0.576	1.0
MBI-GS						
Exhaustion	0.680	−0.366	−0.302	−0.253	−0.292	−0.342
Cynicism	0.443	−0.349	−0.310	−0.260	−0.306	−0.437
Efficacy	−0.226	0.210	0.190	0.160	0.189	0.284

Note. WL: workload. CL: Control. REW: rewards. COM: community. FAIR: fairness. VAL: values. MBI-GS: Maslach Burnout Inventory General Survey. AWS: Areas of Work Life Scale. All correlations were statistically significant (*p* < 0.05).

**Table 6:** Differences in individual parameters of the structural model.

	Sex Group (Male, Female)				Occupation (Teachers, Health Workers)			
Structure Parameter	Δ_b_	Δ_z_	Se	*p*	Δ_b_	Δ_z_	Se	*p*
CL → WL	0.126	0.018	0.18	0.49	0.064	-0.122	0.19	0.74
CL → REW	−0.124	0.041	0.29	0.66	−0.112	−0.141	0.26	0.67
CL → COM	0.180	0.111	0.19	0.36	−0.131	−0.024	0.18	0.46
CL → FAIR	−0.322	−0.215	0.27	0.25	0.195	0.143	0.22	0.39
REW → VAL	−0.126	−0.137	0.13	0.36	−0.368	−0.389	0.16	0.02
COM → VAL	−0.001	0.079	0.17	0.99	0.407	0.332	0.17	0.01
FAIR → VAL	−0.179	−0.097	0.20	0.38	−0.116	−0.069	0.17	0.49
WL → EFF	0.687	−0.154	0.32	0.03	0.504	0.083	0.38	0.19
VAL → EFF	0.214	0.075	0.19	0.27	0.135	0.116	0.20	0.51
EFF → CYN	0.126	0.089	0.12	0.29	0.085	0.157	0.15	0.58
VAL → CYN	0.415	0.166	0.22	0.05	0.456	0.231	0.24	0.06
CYN → EFF	−0.288	−0.346	0.10	0.006	0.176	0.315	0.09	0.07
VAL → EFF	−0.371	−0.285	0.18	0.04	0.079	0.057	0.18	0.67

**Note**. CL: Control. REW: rewards. COM: community. FAIR: fairness. VAL: values. →: representation of effect, for mean beta coefficient. Δ_b_: raw difference in beta coefficient. Δ_z_: Difference Wald test. se: standard error.

## Data Availability

The data used to support the conclusions of this article are not publicly available, but they can be obtained with a reasonable request to the authors.

## Appendix A. Parameters of the MBI-GS in This Study

	**EE**		**EFF**		**Cyn**	
	F	Se	F	Se	F	se
MBI1	0.799					
MBI2	0.801	0.02				
MBI3	0.767	0.02				
MBI4	0.863	0.02				
MBI6	0.886	0.02				
MBI5			0.587			
MBI7			0.666	0.06		
MBI10			0.809	0.07		
MBI11			0.837	0.07		
MBI12			0.742	0.06		
MBI16			0.786	0.07		
MBI8					0.902	
MBI9					0.920	0.020
MBI14					0.745	0.028
MBI15					0.801	0.024
Interfactorial correlations						
EE	1.00					
EFF	−0.421		1.00			
Cyn	0.711		−0.663		1.00	
Note. The parameters are standardized values.						

## References

[1] World Health Organization (WHO) Mental Health at Work. https://www.who.int/news-room/fact-sheets/detail/mental-health-at-work.

[2] World Health Organization (WHO) (2022). Mental Health and COVID-19: Early Evidence of the Pandemic’s Impact: Scientific Brief. https://apps.who.int/iris/bitstream/handle/10665/352189/WHO-2019-nCoV-Sci-Brief-Mental-health-2022.1-eng.pdf?sequence=1&isAllowed=y.

[3] Farooq S., Tunmore J., Ali W., Ayub M. (2021). Suicide, Self-Harm and Suicidal Ideation during COVID-19: A Systematic Review. Psychiatry Res..

[4] Leo C.G., Sabina S., Tumolo M.R., Bodini A., Ponzini G., Sabato E., Mincarone P. (2021). Burnout Among Healthcare Workers in the COVID 19 Era: A Review of the Existing Literature. Front. Public Health.

[5] Vizheh M., Qorbani M., Arzaghi S.M., Muhidin S., Javanmard Z., Esmaeili M. (2020). The Mental Health of Healthcare Workers in the COVID-19 Pandemic: A Systematic Review. J. Diabetes Metab. Disord..

[6] Spoorthy M.S., Pratapa S.K., Mahant S. (2020). Mental Health Problems Faced by Healthcare Workers Due to the COVID-19 Pandemic–A Review. Asian J. Psychiatr..

[7] Ozamiz-Etxebarria N., Mondragon N.I., Bueno-Notivol J., Pérez-Moreno M., Santabárbara J. (2021). Prevalence of Anxiety, Depression, and Stress among Teachers during the COVID-19 Pandemic: A Rapid Systematic Review with Meta-Analysis. Brain Sci..

[8] Silva D.F.O., Cobucci R.N., Lima S.C.V.C., de Andrade F.B. (2021). Prevalence of Anxiety, Depression, and Stress among Teachers during the COVID-19 Pandemic: A PRISMA-Compliant Systematic Review. Medicine.

[9] Bianchi R., Fiorilli C., Angelini G., Dozio N., Palazzi C., Palazzi G., Vitiello B., Schonfeld I.S. (2022). Italian Version of the Occupational Depression Inventory: Validity, Reliability, and Associations with Health, Economic, and Work-Life Characteristics. Front. Psychiatry.

[10] Juárez García A., Gil-Monte P.R., Merino-Soto C., García Rivas J. (2019). Letter to the Editor. From a Polemic Paradox to a Proper Perspective of Job Burnout and Job Satisfaction. J. Neurosurg..

[11] Schaufeli W.B., Leiter M.P., Maslach C. (2009). Burnout: 35 Years of Research and Practice. Career Dev. Int..

[12] Maslach C., Leiter M.P. (2017). Understanding Burnout: New models. The Handbook of Stress and Health.

[13] Leiter M.P., Maslach C. (2009). Nurse Turnover: The Mediating Role of Burnout. J Nurs Manag..

[14] World Health Organization (WHO) ICD-11 for Mortality and Morbidity Statistics. https://icd.who.int/browse11/l-m/en#/http://id.who.int/icd/entity/129180281.

[15] International Labor Organization (ILO) Psychosocial Risks and Work-Related Stress. https://www.ilo.org/global/topics/safety-and-health-at-work/areasofwork/workplace-health-promotion-and-well-being/WCMS_108557/lang--en/index.htm.

[16] World Health Organization (WHO) (2022). Report: World Employment and Social Outlook: Trends. https://www.ilo.org/global/research/global-reports/weso/trends2022/WCMS_834081/lang--en/index.htm.

[17] Siegrist J., Cooper Cary L. (1998). Adverse Health Effects of Effort-Reward Imbalance at Work. Theories of Organizational Stress.

[18] Karasek R.A. (1979). Job Demands, Job Decision Latitude, and Mental Strain: Implications for Job Redesign. Adm. Sci. Q..

[19] Demerouti E., Nachreiner F., Bakker A.B., Schaufeli W.B. (2001). The Job Demands-Resources Model of Burnout. J. Appl. Psychol..

[20] Alsalhe T.A., Chalghaf N., Guelmami N., Azaiez F., Bragazzi N.L. (2021). Occupational Burnout Prevalence and Its Determinants Among Physical Education Teachers: A Systematic Review and Meta-Analysis. Front. Hum. Neurosci..

[21] Shoman Y., El May E., Marca S.C., Wild P., Bianchi R., Bugge M.D., Caglayan C., Cheptea D., Gnesi M., Godderis L. (2021). Predictors of Occupational Burnout: A Systematic Review. Int. J. Environ. Res. Public Health.

[22] Aronsson G., Theorell T., Grape T., Hammarström A., Hogstedt C., Marteinsdottir I., Skoog I., Träskman-Bendz L., Hall C. (2017). A Systematic Review Including Meta-Analysis of Work Environment and Burnout Symptoms. BMC Public Health.

[23] O’Connor K., Muller Neff D., Pitman S. (2018). Burnout in Mental Health Professionals: A Systematic Review and Meta-Analysis of Prevalence and Determinants. Eur. Psychiatry.

[24] Pijpker R., Vaandrager L., Veen E.J., Koelen M.A. (2020). Combined Interventions to Reduce Burnout Complaints and Promote Return to Work: A Systematic Review of Effectiveness and Mediators of Change. Int. J. Environ. Res. Public Health.

[25] López-López I.M., Gómez-Urquiza J.L., Cañadas G.R., De la Fuente E.I., Albendín-García L., Cañadas-De la Fuente G.A. (2019). Prevalence of Burnout in Mental Health Nurses and Related Factors: A Systematic Review and Meta-Analysis. Int. J. Ment. Health Nurs..

[26] Leiter M.P., Maslach C. (1999). Six Areas of Worklife: A Model of the Organizational Context of Burnout. J. Health Hum. Serv. Adm..

[27] Maslach C. (1998). A Multidimensional Theory of Burnout. Theor. Organ. Stress.

[28] Leiter M.P., Maslach C. (2005). A Mediation Model of Job Burnout. Research Companion to Organizational Health Psychology.

[29] Maslach C., Leiter M.P. (1997). The Truth About Burnout.

[30] Leiter M.P., Maslach C., Perrewé P., Ganster D.C. (2004). Areas of Work Life: A Structured Approach to Organizational Predictors of Job Burnout. Emotional and Physiological Processes and Positive Intervention Strategies.

[31] Gascón S., Fueyo-Díaz R., Borao L., Leiter M.P., Fanlo-Zarazaga Á., Oliván-Blázquez B., Aguilar-Latorre A. (2021). Value Conflict, Lack of Rewards, and Sense of Community as Psychosocial Risk Factors of Burnout in Communication Professionals (Press, Radio, and Television). Int. J. Environ. Res. Public Health.

[32] El-Gazar H.E., Abdelhafez S., Zoromba M.A. (2022). Effects of the Areas of Worklife on Job Embeddedness: A National Cross-Sectional Study among Egyptian Nurses. BMC Nurs..

[33] Ostrow L., Cook J., Salzer M., Pelot M., Burke-Miller J. (2022). Predictors of Worklife Burnout among Mental Health Certified Peer Specialists. Am. J. Orthopsychiatry.

[34] Leiter M.P., Maslach C. (2022). Pandemic Implications for Six Areas of Worklife. Burnout While Working.

[35] Apaydin E.A., Rose D.E., Yano E.M., Shekelle P.G., McGowan M.G., Antonini T.L., Valdez C.A., Peacock M., Probst L., Stockdale S.E. (2021). Burnout Among Primary Care Healthcare Workers During the COVID-19 Pandemic. J. Occup. Environ. Med..

[36] Gascón S., Leiter M.P., Stright N., Santed M.A., Montero-Marín J., Andrés E., Asensio-Martínez A., García-Campayo J. (2013). A Factor Confirmation and Convergent Validity of the “ Areas of Worklife Scale” (AWS) to Spanish Translation. Health Qual. Life Outcomes.

[37] Brom S.S., Buruck G., Horváth I., Richter P., Leiter M.P. (2015). Areas of Worklife as Predictors of Occupational Health—A Validation Study in Two German Samples. Burn. Res..

[38] Kitaoka K., Masuda S., Morikawa Y., Nakagawa H. (2015). Japanese Version of the Areas of Worklife Survey (AWS): Six Mismatches between Person and Job Environment. Jpn. J. Adm. Sci..

[39] Nguyen H.T.T., Kitaoka K., Sukigara M., Thai A.L. (2018). Burnout Study of Clinical Nurses in Vietnam: Development of Job Burnout Model Based on Leiter and Maslach’s Theory. Asian Nurs. Res. (Korean Soc. Nurs. Sci.).

[40] Terelak J.F., Izwantowska A. (2009). Adaptacja Kwestionariusza Obszary Życia Zawodowego (Areas of Worklife Survey) Christina Maslach i Michael Leiter. Stud. Psychol. Theor. Prax..

[41] Kaiser H.F. (1970). A Second Generation Little Jiffy. Psychometrika.

[42] Lloret-Segura S., Ferreres-Traver A., Hernández-Baeza A., Tomás-Marco I. (2014). El Análisis Factorial Exploratorio de Los Ítems: Una Guía Práctica, Revisada y Actualizada. An. Psicol./Ann. Psychol..

[43] Matsunaga M. (2010). How to Factor-Analyze Your Data Right: Do’s, Don’ts, and How-To’s. Int. J. Psychol. Res..

[44] Brown T.A. (2015). Confirmatory Factor Analysis for Applied Research.

[45] Reise S.P., Widaman K.F., Pugh R.H. (1993). Confirmatory Factor Analysis and Item Response Theory: Two Approaches for Exploring Measurement Invariance. Psychol. Bull..

[46] Masluk B., Gascón Santos S., Albesa Cartagena A., Asensio Martinez A., Peck E., Leiter M.P. (2018). “Areas of Worklife Scale” (AWS) Short Version (Spanish): A Confirmatory Factor Analysis Based on a Secondary School Teacher Sample. J. Occup. Med. Toxicol..

[47] Dominguez Lara S.A., Fernández Arata M., Calderón de la Cruz G., Juárez García A., Merino Soto C. (2021). Análisis de La Estructura Interna de La Escala de Las Áreas de La Vida Laboral (AWS) En Docentes de Escuelas Públicas de Lima. Rev. Iberoam. Diagnóstico Evaluación Psicológica.

[48] de Winter J.C.F., Dodou D., Wieringa P.A. (2009). Exploratory Factor Analysis with Small Sample Sizes. Multivar. Behav. Res..

[49] Leiter & Maslach Areas of Worklife Survey (AWS)—Assessments, Tests|Mind Garden—Mind Garden. https://www.mindgarden.com/274-areas-of-worklife-survey.

[50] Gjersing L., Caplehorn J.R., Clausen T. (2010). Cross-Cultural Adaptation of Research Instruments: Language, Setting, Time and Statistical Considerations. BMC Med. Res. Methodol..

[51] Beaton D.E., Bombardier C., Guillemin F., Ferraz M.B. (2000). Guidelines for the Process of Cross-Cultural Adaptation of Self-Report Measures. Spine.

[52] Schaufeli W., Leiter M., Maslach C., Jackson S. (1996). Maslach Burnout Inventory-General Survey. The Maslach Burnout Inventory: Test Manual.

[53] Juárez-García A., Merino-Soto C., Fernández-Arata M., Flores-Jiménez C.A., Caraballo M., Camacho-Cristiá C. (2020). Validación Transcultural y Funcionamiento Diferencial Del Maslach Burnout Inventory—General Survey En Docentes de Tres Países Latinoamericanos. Av. Psicol. Latinoam..

[54] The World Medical Association (WMA) (2013). Declaración de Helsinky de La AMM—Principios Éticos Para La Investigaciones Médicas En Seres Humanos. https://www.wma.net/es/policies-post/declaracion-de-helsinki-de-la-amm-principios-eticos-para-las-investigaciones-medicas-en-seres-humanos/.

[55] Yentes R.D., Wilhelm F. (2018). Careless: Procedures for Computing Indices of Careless Responding. R Package Version.

[56] Little R.J.A. (1988). A Test of Missing Completely at Random for Multivariate Data with Missing Values. J. Am. Stat. Assoc..

[57] Rouzinov S., Berchtold A. RBtest: Regression-Based Approach for Testing the Type of Missing Data. R Package Version 1.1. https://cran.r-project.org/web/packages/RBtest/RBtest.pdf.

[58] Tierney N., Cook D., McBain M., Fay C. Data Structures, R Package Version 0.6.1.CRAN—Package Naniar. https://cran.r-project.org/web/packages/naniar/index.html.

[59] van Buuren S., Groothuis-Oudshoorn K. (2011). Mice: Multivariate Imputation by Chained Equations in R. J. Stat. Softw..

[60] Dai S. (2021). Handling Missing Responses in Psychometrics: Methods and Software. Psych.

[61] Merino-Soto C., Juárez-García A., Escudero G.S., Toledano-Toledano F. (2022). Parametric and Nonparametric Analysis of the Internal Structure of the Psychosocial Work Processes Questionnaire (PROPSIT) as Applied to Workers. Int. J. Environ. Res. Public Health.

[62] Keselman H.J., Othman A.R., Wilcox R.R. (2013). Preliminary Testing for Normality: Is This a Good Practice?. J. Mod. Appl. Stat. Methods.

[63] Li C.H. (2016). Confirmatory Factor Analysis with Ordinal Data: Comparing Robust Maximum Likelihood and Diagonally Weighted Least Squares. Behav. Res. Methods.

[64] Maydeu-Olivares A. (2017). Assessing the Size of Model Misfit in Structural Equation Models. Psychometrika.

[65] Shi D., Maydeu-Olivares A. (2020). The Effect of Estimation Methods on SEM Fit Indices. Educ. Psychol. Meas..

[66] Maydeu-Olivares A., Shi D., Rosseel Y. (2017). Assessing Fit in Structural Equation Models: A Monte-Carlo Evaluation of RMSEA Versus SRMR Confidence Intervals and Tests of Close Fit. Struct. Equ. Model. A Multidiscip. J..

[67] Ximénez C., Revuelta J., Castañeda R. (2022). What Are the Consequences of Ignoring Cross-Loadings in Bifactor Models? A Simulation Study Assessing Parameter Recovery and Sensitivity of Goodness-of-Fit Indices. Front. Psychol..

[68] Shi D., Maydeu-Olivares A., DiStefano C. (2018). The Relationship Between the Standardized Root Mean Square Residual and Model Misspecification in Factor Analysis Models. Multivar. Behav. Res..

[69] Nye C.D., Drasgow F. (2011). Effect Size Indices for Analyses of Measurement Equivalence: Understanding the Practical Importance of Differences Between Groups. J. Appl. Psychol..

[70] Zumbo B.D. (1999). A Handbook on the Theory and Methods of Differential Item Functioning (DIF).

[71] Jodoin M.G., Gierl M.J. (2010). Evaluating Type I Error and Power Rates Using an Effect Size Measure With the Logistic Regression Procedure for DIF Detection. Appl. Meas. Educ..

[72] Béland S., Cousineau D., Loye N. (2018). Utiliser Le Coefficient Omega de McDonald à La Place de l’alpha de Cronbach. McGill J. Educ..

[73] DeFreese J.D., Smith A.L. (2013). Areas of Worklife and the Athlete Burnout-Engagement Relationship. J. Appl. Sport Psychol..

[74] Suárez-Alvarez J., Pedrosa I., Lozano L.M., García-Cueto E., Cuesta M., Muñiz J. (2018). Using Reversed Items in Likert Scales: A Questionable Practice. Psicothema.

[75] García-Fernández J., Postigo Á., Cuesta M., González-Nuevo C., Menéndez-Aller Á., García-Cueto E. (2022). To Be Direct or Not: Reversing Likert Response Format Items. Span. J. Psychol..

[76] Arias V.B., Garrido L.E., Jenaro C., Martínez-Molina A., Arias B. (2020). A Little Garbage in, Lots of Garbage out: Assessing the Impact of Careless Responding in Personality Survey Data. Behav. Res. Methods.

[77] Pijpker R., Kerksieck P., Tušl M., De Bloom J., Brauchli R., Bauer G.F. (2022). The Role of Off-Job Crafting in Burnout Prevention during COVID-19 Crisis: A Longitudinal Study. Int. J. Environ. Res. Public Health.

[78] Chirico F. (2016). Job Stress Models for Predicting Burnout Syndrome: A Review. Ann. Ist. Super. Sanita.

[79] Leiter M.P., Maslach C. (2016). Latent Burnout Profiles: A New Approach to Understanding the Burnout Experience. Burn. Res..

[80] Maslach C., Leiter M.P. (2021). How to Measure Burnout Accurately and Ethically. Harv. Bus. Rev..

